# The potential role of adherence factors in probiotic function in the gastrointestinal tract of adults and pediatrics: a narrative review of experimental and human studies

**DOI:** 10.1080/19490976.2022.2149214

**Published:** 2022-12-05

**Authors:** Frida Gorreja, W. Allan Walker

**Affiliations:** a Department of Microbiology and Immunology, Institute for Biomedicine, Sahlgrenska Academy, University of Gothenburg, Gothenburg, Sweden; b Nutrition-Gut-Brain Interactions Research Centre, School of Medical Sciences, Örebro University, Örebro, Sweden; c Mucosal Immunology and Biology Research Center, Massachusetts General Hospital for Children, Harvard Medical School, Boston, Massachusetts, USA

**Keywords:** Probiotic adhesion, immune interaction, IECs interaction, surface structures of bacteria, pathogen inhibition

## Abstract

Numerous studies point to the important role of probiotic bacteria in gastrointestinal health. Probiotics act through mechanisms affecting enteric pathogens, epithelial barrier function, immune signaling, and conditioning of indigenous microbiota. Once administered, probiotics reach the gastrointestinal tract and interact with the host through bacterial surface molecules, here called adhesion factors, which are either strain- or specie-specific. Probiotic adhesion, through structural adhesion factors, is a mechanism that facilitates persistence within the gastrointestinal tract and triggers the initial host responses. Thus, an understanding of specific probiotic adhesion mechanisms could predict how specific probiotic strains elicit benefits and the potential of adherence factors as a proxy to predict probiotic function. This review summarizes the present understanding of probiotic adherence in the gastrointestinal tract. It highlights the bacterial adhesion structure types, their molecular communication with the host and the consequent impact on intestinal diseases in both adult and pediatric populations. Finally, we discuss knockout/isolation studies as direct evidence for adhesion factors conferring anti-inflammatory and pathogen inhibition properties to a probiotic.

**What is known**:

- Probiotics can be used to treat clinical conditions.
- Probiotics improve dysbiosis and symptoms.
- Clinical trials may not confirm *in* *vitro* and animal studies.

**What is new**:

- Adhesion structures may be important for probiotic function.
- Need to systematically determine physical characteristics of probiotics before selecting for clinical trials.
- Probiotics may be genetically engineered to add to clinical efficacy.

## 1. Introduction

The human microbiota is a complex community that makes major contribution to human health.^1,2^ The gastrointestinal tract (GIT) microbiota has been referred to as an ancillary “organ” due to its impact on human well-being, including host metabolism, nutrition, physiology, and immune function.^3^ The GIT microbiota harbors a complex microbial community, including prokaryotes, eukaryotes, and archaea. Several comprehensive microbial studies have focused on identification of individual organisms in this community.^4–7^ However, identification alone does not explain which specific part of the “organ” is functionally responsible for human health benefits.

Probiotics have been shown to affect the human GIT and microbiota from birth when administered to neonates.^8^ They are currently available as food supplements, either as prokaryotic probiotics (bacteria) or eukaryotic probiotics (yeast). Prokaryotic probiotics, typically belong to the genera *Lactobacillus* and *Bifidobacteria*, are most commonly used in treating disease.^9^ Selecting probiotics from these genera with potential health benefits was initially studied using in vitro models such as intestinal epithelial cells (IECs), usually Caco-2 cell lines,^10^ and animal models.^11^ These models are used to screen for candidate strains isolated from food, humans, or animals as well as to investigate the mechanism of action of probiotics on the GIT. However, despite the final goal to translate knowledge gained from in vitro into human studies, the complexity of the human GIT hampers this transition. While *in* *vitro* experiments help to select a potentially effective strain, and animal studies may demonstrate efficacy, human clinical studies that can confirm health effects often fail.^12,13^

The possible reasons for human clinical studies failing to confirm probiotic effectiveness reported *in* *vitro* and animal studies are under discussion. One of the reasons is thought to be inefficient adherence of probiotics. Intrinsic molecular and structural characteristics of both bacteria and human hosts affect probiotic adherence. For example, oligo- or poly-saccharide structures, appendages and specialized surface proteins on bacteria interact with the host to induce innate immune signaling^14^ or prevent pathogen attachment.^15^ Some of these structures, such as pili on LGG (*Lacticaseibacillus rhamnosus* GG, former^16^ *Lactobacillus rhamnosus*), seem to be well characterized.^17^ However, there is no clear-cut association between adhesion structures and health parameters reported in literature.

Adhesion of probiotics is defined as an initial bond or grip of probiotic bacteria and is based on unspecific physical interactions to a certain surface. Adhesion factors or adhesins are the molecular structures on the surface of the probiotic bacteria that facilitates this bond. This initial adhesion then initiates distinct interactions between adherence structures on bacteria and e.g. corresponding receptors on the host.^18^ Some researchers have defined some of these adhesion factors as “cell-derived components”.^19^ Adhesion of probiotic bacteria to GIT is considered a key aspect in relation with the host immune system modulation as well as for the exclusion of enteric pathogens.^20–22^ Furthermore, an increasing number of studies are demonstrating that the viability of bacterial cells is not essential to exert immunomodulatory effects but rather their isolated adhesins can perform as well as when present as part of the bacterium.^23^ Hence, in this review, we consider as adhesion factor both the adhesin in situ on the probiotic bacterium and the isolated/purified adhesin.

To establish whether a potential health benefit is shaped by probiotics, more attention is being focused on mechanistic knowledge, such as how do adherence mechanisms affect probiotic function.^24^ For instance, adhesion of probiotics may be important for probiotic replication and colonization (Box 1), immunomodulation, inhibition of pathogen colonization. This direct interaction involves GIT immune and epithelial cells, as well as resident commensals. Hence, determining adhesion conservancy within the GIT can potentially be considered to predict probiotic function in humans.^25^

Box 1.

**Table:** 

The term *”*colonization*”* used in this review refers to temporary presence and replication of the administered bacteria.^26^ In addition, colonization is investigated tracing both (e.g. through stools) the administered bacteria and the strains (beneficial, pathogenic) whose abundance is affected by the administered bacteria. The presence or absence of probiotics in the stools is very strain-specific and it depends on the composition of the indigenous microbiota which can favor longer persistence for certain individuals in healthy and diseased GIT.^27^ This highlights the importance of proper dose selection and sample size when designing clinical trials evaluating probiotics. Probiotics can directly or indirectly affect colonization of other bacteria.^28^ All these direct and indirect mechanisms consequently facilitate survival and replication or bacteria that are associated with health. Direct influence by probiotic is provided by production of e.g. antimicrobial peptides, short chain fatty acids or/and nutrients. In addition direct binding to immune cells can trigger immune responses against specific bacteria. The later is less studied for specific probiotics. Indirectly probiotics can impact colonization by stimulating mucin production, hence adherence. Other consequente downstream effects, mentioned throughout the review, are also anti-inflammation mechanism, reinforcement of intestinal barrier function (e.g. tight junctions on IECs). Once probiotic bacteria are administered the extent to which they colonize has been shown to depend on:^29^ The age group of individuals under study: infants, children, adults, elderly. Frequency of administration: few single doses (e.g. in acute diarrhea) vs short term (few weeks) vs long term administration (several weeks). *D*ose: whether considered high or low dose is dependent on the individual studies for specific strains. Dose choice is for the most driven by the age of the individuals, condition of the GIT, and dose identification by *in vitro* screening of the probiotic. Colonization, in the sense of long-term persistence, can be considered a potential safety issue and however permanente colonization is rare.^30^

The purpose of this review is to assess the surface structure of probiotics that are potentially responsible for adhesion. A review from Javanshir et al.^31^ and a mini-review Monteagudo-Mera et al.^32^ provide another perspective on the importance of the topic. Although the reviews focus more on the host perspective and some bacterial perspectives, more reviews are needed to supplement. We provide an additional overview of how probiotics can interact with the human host and suggest health implications deriving from such interactions. We focus on classes of bacterial surface structure molecules and consider the role they may play in GIT adhesion. Examples of pre- and clinical evidence are provided. Finally, we consider studies where these adhesion factors have been knocked out or purified to show their direct impact on probiotic function.

## 2. Adhesion Factors And Potential Downstream Mechanisms

### 2.1 Probiotic bacteria surface molecules involved in adhesion

**Figure 1.**
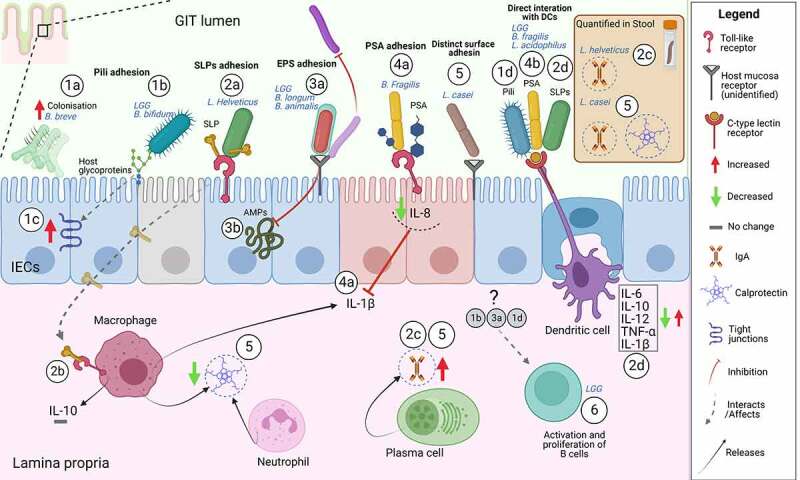
**Examples of mode of action of probiotic adhesion factors**. The figure shows binding and downstream effects on intestinal epithelial cells (IECs) and immune cells, reported for some probiotics with characterized adhesins. Probiotic pili-mediated adhesion favors a better colonization^34^ (1a), direct interaction to the intestinal mucus glycoproteins^35^ (1b), increased expression of tight junction-encoding genes^36^ (1c) and direct interaction to antigen-presenting cells (dendritic cells)^37^ (1d). SLPs-mediated adhesion through SLPs on bacteria or isolated SLPs occurs through interaction with TLR receptors on IECs (2a) and immune cells (macrophages)^23,38,39^(2b) possibly this sustains production of fecal IgA^40^(2c). Studies using bacterial mutants of either SLPs or SLPs co-localized molecules show that SLPs trigger antigen-presenting cells (dendritic cells) inducing pro-inflammatory (IL-12, TNF-α, IL-1β) and anti-inflammatory cytokines (IL-6, IL-10)(2d).^41–43^ Probiotic EPSs-mediated adhesion directly on other bacteria and/or competition for common adhesion sites on the host promotes “competitive exclusion” of pathogens or interferes with probiotic adhesion (3a).^44,45^ EPSs were shown to protect from innate immune mechanism via AMPs the probiotic LGG after adhesion to IECs^46^ (3b). PSA on *B. fragilis* or isolated PSA has been shown to inhibit IL-1β induced inflammation through interaction with TLR2 and 4 on IECs^47,48^ (4a) and can also directly bind to antigen-presenting cells (dendritic cells) triggering downstream immune responses^49,50^(4b). Distinct adhesins in the bacteria cells, not belonging to any of the aforementioned class, could support the sustained effects of *L. casei* on IgA and calprotectin ^51–54^(5). LGG probiotic triggers immediate adaptive immune response via B cells in humans.^13^ Although the responsible upstream adhesion factors are not known the immediate effect is most possibly due to adhesion factors (6). The illustrative figure is shown without subdivision of GIT location (i.e. small or large intestine) but regional differences in the GIT immune system are of utmost importance.^55^ In addition, the figure does not represent all mechanisms investigated by the studies in this review or literature but is meant to illustrate examples. Abbreviations: IECs, intestinal epithelial cells; PSA, polysaccharide A; SLPs, surface layer proteins; EPS, exopolysaccharides; IL, interleukin; IgA, immunoglobulin A; LGG, *Lacticaseibacillus rhamnosus* GG; AMPs, antimicrobial peptides. Created with BioRender.com.

Probiotics interact with the GIT mucosa through their bacterial surface structures. These structures are thought to facilitate adhesion and therefore contribute to probiotic persistence in the GIT mucosa. Consequently, surface structures may enhance probiotic function. In addition, adhesion is of major importance for probiotic colonization (Box 1) inside the host. Hence, adhesion to IECs *in* *vitro* is one of the leading selection criteria to determine whether a bacterial strain can potentially be a probiotic.^20^ Traditionally adherence has been associated with the infectious mechanism of pathogenesis. While characterization of adhesion mechanisms and structures are ongoing for probiotic bacteria their surface structures resemble to a certain extent those of pathogens.^33^ The surface structures on known probiotics are also referred to as adhesins, adhesion factors or ligands. Examples are exopolysaccharides (EPSs), pili and distinct surface proteins.^17^ We discuss below and in Figure 1 some examples of adhesins and downstream interaction mechanisms.

### 2.2 Exopolysaccharides

Exopolysaccharides (EPSs) are polymers present on the surface of the bacterial cell wall, hence the prefix exo-, composed of polysaccharide structures.^56^ EPSs are present on many bacteria, including *Lactobacillus* and *Bifidobacterium* genera.^44,56–58^ Functionally, EPSs enable communication between host cells and colonizing probiotics. EPSs support probiotic survival by adhering to GIT mucosal epithelium.^59–61^ Hence, EPSs are involved in host interactions resulting in bacterial tolerance to harsh GIT conditions,^58^ immunomodulatory activity^46^ and have a role in biofilm formation.^56,59^ The first study to report the effect of probiotic EPSs as bacterial adhesins was a study assessing the EPSs function on LGG, *Bifidobacterium longum* NB667, and *Bifidobacterium animalis* IPLA-R1.^44,62^ The study showed that there is a dose-dependent effect of EPS that interfered with the adhesion of probiotics and pathogens to human intestinal mucus (Figure 1).^44,62^ Since then further studies have investigated the EPSs of these three bacteria. The probiotic LGG seem to use EPSs to interfere with adherence of pathogens in a dose-dependent manner.^44–46^ Similarly, for EPSs in *B. animalis* IPLA-R1^44^ and *B. animalis* subsp. lactis^63,64^ it was shown a variable interference with other bacteria (probiotics and pathogen) via probiotic specific EPSs or biofilm formation. In conclusion, EPSs are thought to bind to external bacterial structures in both pathogens and commensals. Hence the bound EPS blocks the bacteria from adhering to the intestinal mucus.^44^ This impact of probiotics on pathogens was named “competitive exclusion” and suggests the importance of EPS adhesins in this probiotic function in adults and neonates.^44,65,66^

The EPSs on *Bifidobacterium breve* UCC2003 reduce the production of pro-inflammatory cytokines and immune cells.^67^ EPSs help this probiotic to remain immunologically silent while exerting other functions such as pathogen exclusion. In addition, EPSs allowed this probiotic to be tolerated and to interfere with the persistence of the pathogen *Citrobacter rodentium*.^67^ *Citrobacter rodentium* is a model used to investigate human GIT diseases (*E. coli* infections, inflammatory bowel disease, GIT tumors) hence interference with this pathogen could have implications for GIT chronic diseases.^68^ Other *B. breve* strains, Bb99 and BBG-001, have been investigated in RCTs in infants. Bb99 was shown to modify beneficial microbiota in infants treated with antibiotics or delivered through cesarean section.^69^ However, BBG-001 administration failed to protect from necrotizing enterocolitis (NEC) and sepsis in preterm babies.^70^ NEC is a frequently encountered devastating condition in the premature neonates with symptoms similar to sepsis but differentiated by pneumatosis and portal venous gas.^71^ Overall, although the adhesion factors of *B. breve* strains BBG-001 and Bb99 are not well investigated, the outcome of several studies suggests a direct effect of *B. breve* strains on other bacteria (pathogens, commensals) hence indirectly on the mucosa.

### 2.3 Glycolytic enzymes

Administration of *Lactiplantibacillus plantarum* HEAL9 (former^16^ *Lactobacillus plantarum*) and *Lacticaseibacillus paracasei* 8700:2 (former^16^ *Lactobacillus paracasei*) are thought to modulate the peripheral immune response in children with celiac disease autoimmunity.^72^ This effect is enhanced by the ability of these two strains to attach to the human mucosa through a mannose-binding adherence mechanism.^73,74^ Interestingly, *L. plantarum* HEAL9 is genetically similar to the well-studied strain *L. plantarum* 299 v that could hypothetically behave in a similar manner.^72,75^ *L. plantarum* 299 v might adhere through multiple glycolytic enzymes.^74,76^ Although the contribution of each of these structures is difficult to study, because the mutants of this strain lose the metabolic activity conferred by these structures.^76^ Administration of *Lactiplantibacillus plantarum* IS-10506 increased fecal immunoglobulin A (IgA).^77^ IgA is an important mucosal humoral immunity antibody. The probiotic increased the immune response in two different clinical trials in children greater than two years of age and pre-school children.^77,78^ Once in contact with IECs, *L. plantarum* IS-10506 can adhere with its cell wall lipoteichoic acid and peptidoglycan.^77,78^ This adhesion potentially triggers signaling between IECs and nearby plasma cells which are the main producers of IgA.^78^

### 2.4 Encapsulation

Bacterial encapsulations contribute directly and indirectly in the probiotic mechanisms of adhesion. Direct contribution is provided by engaging specific capsular components, such as polysaccharide A described in detail below, that interacts with IECs and immune cells. Indirectly, encapsulation confers probiotics the ability to persist and colonize longer in the GIT (Box 1). As such example, probiotic *Bacillus coagulans* Unique IS2, has been shown to be beneficial for children with irritable bowel syndrome (IBS).^79^ This effect is due to strengthening of intestinal barrier function and reduction in bowel hypersensitivity. As opposed to other probiotic species, *B. coagulans* is a naturally encapsulated spore-forming bacterium. This encapsulation makes the bacteria potentially more viable and resistant to heat and acid degradation (gastric and bile acid). Hence, when administered it reaches the distal bowel and colon, mostly unaffected, where IBS symptoms appear.^79^ Similar effects were observed in an RCT in adults suffering with IBS.^80^ In addition, in this second study, with treatment, no changes were detected in pro- and anti-inflammatory cytokine levels suggesting that *B. coagulans* Unique IS2 could exert its effect by interacting with IECs and microbiota rather than a direct contact with innate immune cells.^80^ It is important to note that adhesion of probiotics regards both that of bacteria cell (probiotic) to host cell and bacteria cell (probiotic) to bacteria cell (microbiota) with the second being defined either auto-aggregation or coaggregation.^81^ Hence, benefits of encapsulation, with respect to adhesion and biofilm formation, are being widely explored to produce bioengineered probiotics mixtures. Probiotics such as *Bacillus subtilis* contain a natural extracellular matrix that surrounds the bacteria and facilitates their attachment to surfaces, hence can support their own and other bacterial survival to harsh industrial and GIT conditions.^82,83^

#### 2.4.1 Polysaccharide A

Numerous gram-positive bacteria synthesize surface polysaccharides such as teichoic acids or lipoteichoic acids, which are important in the adherence of bacteria to biological surfaces. Polysaccharide A (PSA) is the immunodominant capsular polysaccharide of the human symbiont *Bacteroides fragilis* NCTC 9343. PSA is identified and isolated from the *B. fragilis* NCTC 9343 capsule and shown to be an important adherence factor communicating with the intestinal innate and adaptive immune cells of the neonatal and adult host.

First, bacteria containing PSA as well as purified PSA display the same anti-inflammatory properties through direct interaction to receptors on immature IECs as part of innate immune responses.^48^ We have shown in our experimental studies, using an *in vitro* human fetal model, that PSA inhibits IL-1β-induced inflammation through toll-like receptors 2 (TLR2) and 4 on IECs.^48^ In addition, our novel study described the anti-inflammatory role of Zona pellucida protein 4 (ZP4).^47^ ZP4 is a distinctive protein on immature fetal IECs that mediates PSA anti-inflammation effects by involving TLR2 and IL-8^47^ (Figure 1). IL-1β is a pro-inflammatory cytokine released mainly from intestinal macrophages during cell activation and acute inflammation. Elevated cytokines such as IL-1β and IL-8, inhibited by PSA, are important because they have been associated with the diagnosis of necrotizing enterocolitis (NEC).^84^ Hence, inhibition of IL-1β-induced intestinal inflammation by bacterial PSA is strategic for NEC prevention. Such bacterial components, that do not require a live bacterium and are able to maintain immunogenic effects, could potentially be introduced in formula for pre-term infants. Using bacterial components rather than using whole bacterial probiotics could potentially trigger a fine-tuned immune reaction, given the nature of the premature intestine.

Second, PSA binds directly to a C-type lectin receptor that is a glycan-binding receptor on dendritic cells (Figure 1) and this interaction is crucial for its processing and presentation to T cells as part of adaptive immune responses.^50,85^ PSA forms a complex with major histocompatibility complex class II of innate immune cells and is presented to the T cell receptor of CD4^+^ T cells. This antigen presentation mechanism was believed to apply exclusively to protein antigens.^86^ However, PSA purified from *B. fragilis* NCTC 9343 was shown to protect animals from experimental colitis through induction of anti-inflammatory IL-10 producing CD4^+^ T cells.^49^

Summarizing, PSA on *B. fragilis* has been studies in animals and in vitro in humans using inflammatory models of innate and adaptive immune system to try to prevent colitis/NEC/inflammation in adults and neonate models. PSA is an important and promising adhesion factor as part of the capsule of this bacterium with immunogenic properties. Although experimental data are promising, human studies are lacking and of great need for further confirming the application of such purified factors in nutritional recommendations.

### 2.5 Pili appendages

Pili, as probiotic appendages, consist of a protein called pilin, with the capacity to adhere to other bacteria mediating biofilm formation and bacterial aggregations, and to adhere to GIT surfaces mediating the probiotic interactions with the host. Various types of pili structures have been identified on both gram-positive and gram-negative bacteria and have received attention since the LGG probiotic comparative genomic study in 2009.^35^ Since then, a immense number of studies, discussed in detail below, have investigated the pili of LGG.^17,87–92^ In addition to LGG, other bacteria also contain functional pili such as *Bifidobacterium bifidum* PRL2010,^93^ *Bifidobacterium breve* UCC200,^34^ *Lactococcus lactis* IL1403 and TIL448,^94,95^ *Lactobacillus ruminis* ATCC 25644^96^ and *Lacticaseibacillus casei* LOCK 0919 (former^16^ *Lactobacillus casei* LOCK 0919).^97^

#### 2.5.1 Sortase-dependent pili on *B.* *bifidum*

*Bifidobacterium bifidum* PRL2010, a strain isolated from infant stools, is shown to have sortase-dependent pili proteins (Figure 1). Pili conferred to this probiotic both adhesion properties to IECs and immunomodulatory properties *in* *vivo*. This was demonstrated by expressing the same coding sequence of the pili on a second bacteria without pili that manifested the same pili-conferring properties.^93^ *Bifidobacteria* strains are predominant species colonizing the infants GIT and commensals themselves express common adhesion extracellular proteins with *B. bifidum* PRL2010. Investigating probiotic properties of bacteria isolated from healthy infant stool is important for designing pediatric GIT disease treatments where such strains could colonize and re-establish symbiosis. Effects of *B. bifidum* PRL2010 on intestinal barrier function, IECs and innate immune responses was confirmed by other studies.^36^ These effects were achieved through the transcriptional regulation of tight junction genes for induced colitis in mice.^36^ Hence, the piliation of *B. bifidum* and other pili containing probiotics is viewed with an expanded role as a niche‐adaptation factor.^98^

#### 2.5.2 Sortase-dependent pili on LGG

The probiotic LGG, one of the most frequently administered probiotics in adults and children, exert some of its probiotic properties via a well characterized pili adhesion factor. LGG contains two separate pilus gene clusters in its genome, SpaCBA and SpaFED.^17,35,99^ However, SpaCBA pilus is functionally important for the well-organized adherence of this probiotic.^35,87,99^ The functional pilin motifs SpaCBA on LGG contains 3 type of pilins.^35,99,100^ These pilins are monomeric protein subunits joined together covalently by a pilus-specific sortase enzyme hence called sortase-dependent pilus type.^101^ LGG employs this structure to assure strong adherence to glycoproteins on the intestinal mucus, colonization and functional probioticproperties.^17,35,87,99^ (Figure 1) (Box 1). It was shown that acid stress could also enhance the GIT adhesion capability of LGG by inducing pili-related genes on the bacterium.^102^

Upon adhesion, LGG pili are important adhesins with immunomodulatory properties on the intestinal mucosal.^13,103^ In fact, glycans on LGG pili can be recognized by dendritic cells via a C-type lectin receptor^37^ (Figure 1). These interactions are of functional importance to induce dendritic cells hence pro-inflammatory cytokines IL-6 and IL-12 and anti-inflammatory cytokine IL-10^37^ (Figure 1). While this innate immune signaling can be induced also by several commensals, to what extent this relates to adaptive immunity is not known. Bornholdt et al^13^ showed that only 2 h after administration of LGG, it was found in the jejunum of healthy participants. The study clustering analysis shows that adaptive immunity (B-cell activation) genes were upregulated only in one third of the participants.^13^ This suggests that individual differences should be taken into account when designing human studies. Interestingly, the effects on adaptive immunity were immediate as B-cell gene changes were detected 2 h after LGG administration. In addition, another human study with a longer administration time (28 days) found transcriptional changes (in blood) of immune cell trafficking and inflammatory responses.^103^ Expression was restored after the probiotic stopped, suggesting that the long term effect was due to the probiotic.^103^ Although subjects in this study were healthy, induction of immune cells by LGG, if administered to inflamed intestine, could potentially maintain immunological tolerance while exerting anti-inflammatoryeffects.^13,103^ Finally, the effects of the pili on innate immune components, such as dendritic cells, could potentially render this probiotic useful in inflammatory conditions where this cells play key roles in diseases pathogenesis^104,105^ (Figure 1).

Studies have also provided insights into the role that LGG can play in the host intestinal barrier function.^106–108^ Intestinal barrier function is commonly studied using IEC models such as Caco-2 and by investigating tight junction expression (Zonula occludens-1, occludine, claudins) which are proteins holding together IECs hence keeping tight the intracellular passage of substances in the GIT. Hence, *in* *vitro* experimental models employ disruption of these tight junctions as a proxy for GIT disease models. LGG prevented interferon-gamma-induced epithelial barrier disruption used as a model for IBS. Barrier function via Zonula occludens-1 and occludine was protected in IBS-like enteroids but such protection was lost when using denatured LGG suggesting that pili might need viable bacteria to exert its function.^106^ A similar protective effect of LGG was observed in a gliadin disrupted barrier function as a model for celiac disease. Only viable LGG in concomitant treatment with gliadin significantly increased Zonula occludens-1, claudin-1 and occludine gene expression in IECs Caco-2.^107^ In conclusion, LGG protects intestinal barrier function and this protection is lost when employing not-viable bacterium. Differently to other adherence factors, there are no studies yet showing whether is possible to purify and use pili. However, LGG mutants are being engineered to adjust pili expression on bacteria mutants.^109^ This anticipates a future of specialized mutants with varied capability to adhere.

### 2.6 Distinct surface adhesins

Some probiotics can have diverse surface adhesins, which are not associated with any of the abovementioned categories. These distinct adhesins have been considered in isolated strains with probiotic activity.^110^ They are often of proteinaceous nature such as the Family 1 of solute binding proteins on *B. infantis*, a common member of infant intestinal microbiota.^111,112^ Adhesion factors of proteinaceous nature, defined here as distinct proteins, can perform more than one function but are also involved in adhesion. Groups of proteinaceous adhesins are for instance surface layer proteins (SLPs), collagen binding protein (Cbp), mucus-binding proteins (Mub), mucus adhesion promoting protein (MapA), sortase A, auto-aggregation promoting protein (AggLb). We will consider some of these in detail below.

#### 2.6.1 Surface layer proteins

Surface layer proteins (SLPs), or S-Layer proteins, are a class of proteins that form the outermost interacting component of the bacterial cell wall of different *Lactobacillus* species.^113,114^ Presence of SLPs appendages is beneficial especially for *Lactobacilli* probiotic immunomodulatory action in the GIT. SLP and its homologue on *Lactobacillus acidophilus* NCFM affects immune response via dendritic cells (Figure 1) and interaction with IECs.^41,42,115^ SLPs on *L. acidophilus* NCFM and ATCC 4365 are functionally involved ligands that interact with a C-type lectin receptor on dendritic cells and thereby prime these cells to regulate T cell function.^41,43^ *L. acidophilus* NCFM has been especially well-studied. Several type of SLPs have been identified for this probiotic (e.g. SlpA, SlpB).^116^ It was shown, by using SLP mutants, that the type of SLP on *L. acidophilus* NCFM controls the cytokine type production.^41,115,117,118^ Compared to other probiotic *Lactobacilli, L. acidophilus* NCFM displays a slight proinflammatory profile with a very low IL-10/IL-12 cytokine ratio that has been directly linked to SLPs/SLP associated proteins.^41,115,117,118^ However, it is not clear to what extent each of these SLP protein types within *L. acidophilus* NCFM contributes to probiotic properties. The difficulty to study this is given by the fact that the knockdown of one protein has resulted in the changes in expression of another making it hard to attribute a single effect to a single SLP protein. ^41,115,117,118^ SLP immunological properties are highly variable between different *Lactobacilli* as well despite their purified SLPs inducing the production of IL-12 p40 on macrophage cell line THP-1 from multiple strains.^118^

Adhesion and colonization of *Ligilactobacillus salivarius* REN (former^16^ *Lactobacillus salivarius* REN) to human IECs is mediated by an S-layer protein called choline-binding protein A (CbpA).^119^ The interaction was shown *in* *vitro* to be mediated via an enolase receptor on IEC HT-29 cells that can recognise CbpA.^119^ It is not further documented how CbpA affects this probiotics properties but is interesting to know that detailed characterisation of each single *Lactobacilli* SLPs are starting to be elucidated.

#### 2.6.2 Collagen-binding protein

Collagen-binding proteins (Cbp) are cell surface proteins on bacteria able to bind and adhere to GIT extracellular matrix components including collagen. This process was identified initially as pathogenic as it allowed colonization of pathogens once firmly bound to collagen, however probiotics can mimic the same mechanism without causing harm. Probiotic *Lactobacillus* strains, including *Lactiplantibacillus plantarum* LM3, 91 and W2 are able to bind to collagen.^120–122^ Purified Cbp from *L. plantarum* 9, a strain selected for strong collagen binding among several *L. plantarum*, displays competitive exclusion (anti-adhesion) properties on pathogenic *Escherichia coli* 0157:H7.^123^ Presence of adhesion factors like Cbp confer impactful colonization potential to probiotics under the harsh environment of the GIT and given that collagen is a component of mucus it is easily accessible.^123,121^ *L. plantarum* W2 was able to inhibit pathogen *Penaeus vannamei*. This probiotic cntains a Cbp in its genome although the study did not attribute the probiotic property solely to the Cbp.^122^

*Lacticaseibacillus casei* supplementation during acute diarrhea in children of 6 months-6 years increased fecal IgA and reduced fecal lactoferrin and calprotectin^52^ (Figure 1). Induction of IgA deposition by this probiotic seems to be continuous and sustained.^51^*L. casei* ATCC 393 has distinctive adhesion properties, although minimum adhesion to confer probiotic properties is observed in the large intestine it is comparable to other probiotics.^53^ It is not clear form literature which adhesin is characteristic for which *L. case* strain. However, attempts have been made to genetically modify this probiotic to expresses collagen-binding protein gene *cnb*, which in turn enhances bacterial adhesion.^54^

In conclusion, Cbp seems to naturally benefit *L. plantarum* strains to colonize better by adhering to GIT collagen. There seems to be some potential for pathogen inhibition, possibly due to common collagen-binding sites shared by probiotics and pathogens, but the studies are merely focusing on the bacteria and don’t have clinical implications yet. Interestingly, Cbp is purifiable and as such future research could consider administration of Cbp as adjuvant to infections without the complexity of assuring viable probiotics.

#### 2.6.3 Mucus-binding or mucus adhesion proteins

Mucus-binding proteins (Mub) and mucus adhesion proteins (MapA) are the two key cell surface proteins expressed differentially among species of *Lactobacilli*, thus, promoting their attachment to GIT mucosa.^124^ Mab and MapA mainly bind to mucins which represent the majority of proteins in the mucus of GIT. The nomenclature and classification of specific Mub and MapA, implicated in probiotic adhesion, is unclear from literature. In general, comparative genomic studies have assigned Mub and MapA names to genes or proteins identified in specific probiotic strains that can bind to GIT mucus components, usually of glycan nature. Mub is found in the surface of the bacterial cell and can contain mucin-binding repetitive domains (MucBP) that are functionally responsible for the interaction with the mucus.^125^

*Limosilactobacillus reuteri* strains (former^16^ *Lactobacillus reuteri*) contains both Mub and MapA adhesion factors. Mub from *L. reuteri* 1063 was able to interact with mammalian Igs including IgA which is important for GIT mucosa homeostasis, and interacted also directly with GIT mucus.^126,127^ Similarly, a Mub on *L. reuteri* ATCC PTA 6475, named CmbA, was responsible for adhesion in IECs Caco-2. After evaluation of 5 potential adhesins this study observed strong loss of adhesion mainly in *cmba* bacteria mutants.^128^ *L. reuteri* ATCC 53608, as an architype of a commensal bacteria, has 14 tandemly arranged Mub repeats and a motif called LPXTG that anchors to the bacteria cell side.^129^ Mub of this bacteria was shown to be an adaption niche that organizes in a way to maximize the adhesion to GIT mucus glycans.^129^ Both Mub on *L. reuteri* ATCC PTA 6475 and 53,608 have been shown to be able also to trigger an immune response and inhibition of *E. coli*. First, immunoregulatory properties are exerted via interaction of C-type lectin receptors on dendritic cells and hence influence production of both anti- and pro-inflammatory cytokines IL-10, TNF-α, IL-1β, IL-6, and IL-12.^130^ Second, pathogen inhibition properties on enteropathogenic *E. coli* were shown both in mucus producing and non-mucus producing IEC lines as well as small intestine tissue.^131^

Several studies have investigated the role of MapA as well on the probiotic strains *L. reuteri*. MapA on *L. reuteri* 104 R is considered a primary adhesion factor for adhesion of this probiotic in IECs and mucus.^132^ This was shown by pre-treatment with purified MapA that bound to multiple receptor-like structures on IEC Caco-2 cells and subsequently inhibited *L. reuteri* in a dose-dependent manner showing a saturation of the receptors.^132^ Parts of MapA, as a larger surface structure on *L. reuteri* LA92, were defined for their antimicrobial peptide properties and named AP48-MapA.^133^ Such pleiotropic functions of MapA, both as GIT adhesion factor and for antimicrobial peptide properties, was proposed to be of major importance in establishing a healthy microbiota.^133^ While *L. reuteri* 104 R and LA92 are not currently used in the clinical settings, *L. reuteri* DSM17938 is approved for human use to treat infant diseases. However, no consensus has been reached, as reported by meta-analysis, on the benefit of this strain, especially for formula-fed infants with colic.^134^ In various RCTs using *L. reuteri* DSM17938, this probiotic failed to show efficacy alone, or compared to placebo (or control) treatment of acute diarrhea in infants.^135^ For constipation, the results were contradictory in children aged 3–7, 2–16 and 2–4 years.^136–138^ This lack of clarity could be attributed to differing administration length, doses of probiotic or to insufficient patient numbers. While Mub and MapA have not yet been investigated for DSM17938, their presence seem to be an advantage for several *L. reuteri* and worth investigating.^139^ This was shown by the presence of MapA genes in eight tested *L. reuteri* that increased significantly upon co-culturing with Caco-2 cells as a model for intestinal barrier function.^139^ Finally, the presence of Mub and MapA suggests opportunities for new *L. reuteri* strains to be introduced as potential probiotics.

*L. plantarum* 91 exhibits strong probiotic traits such as acid and bile tolerance and colonization, that *in vivo* were partially attributed to Mub genes which increased expression during the bacteria transit in the stomach of mice.^124^ The Mub of *L. plantarum* 91 has 6 mucus-binding domains where the last 2 domains of the Mub are considered functionally responsible, named Mubs5s6, and exhibit high adhesion in human GIT tissue. It has been possible to purify Mub from *L. plantarum* 91 and further express it in other species.^140^ Pre-treatment with purified Mubs5s6 of IEC lines Caco-2 and HT-29 inhibited the binding of enteropathogenic *E. coli*. This mechanism was attributed to the binding of Mubs5s6 to cytokeratins, Hsp90, and Laminin (all three ligands associated with infections) in the host mucosa. The effects of purified Mubs5s6 were potentially stronger than the effects of bacteria cells expressing Mubs5s6.^141^Another *L. plantarum, L. plantarum* 423, showed putative probiotic genetic characteristics given by the presence of Mub, MapA and adhesion-like factor EF-Tu.^142^ Gene expression changes of Mub, MapA and EF-Tu were evaluated in the presence of mucus, bile, pancreatin, different pH, and it was shown that the probiotic can adapt to such conditions of a healthy GIT simulation.^142^

In conclusion, Mub and MapA seem to be very important adhesion factors for several different *Lactobacilli* that share common niche genes. Although adhesion on mucus-bearing or non-mucus bearing IECs and endurance of GIT conditions seem to be strongly guided by Mub and MapA presence, the clinical significance of such adhesion factors is still blurry. One interesting observation is that of the enteropathogenic *E. coli* inhibition both by Mub bearing strains as well as purified Mub. *E. coli* pathogenic strains are involved in sudden infant death.^143^ The inhibition of such strains by purified Mub and Mub bearing *Lactobacilli*, possibly by the pathogen and probiotic sharing common adhesion sites, opens new frontiers in designing prevention treatments for at risk infants.

#### 2.6.4 Multiple adhesion factors

Genomic analysis of commercially available *Bacillus clausii* ENTPro, revealed three proteins involved in adhesion: mucus-binding protein, a collagen-binding protein and a fibronectin-binding protein functionally responsible for adhesion.^144^ The study also proposes that probiotic strains within *B. clausii* (i.e ENTPro, B106, and UBBC-07) are very similar to each other in this regard.^144^ *B. clausii* UBBC-07 supplementation resulted clinically in improving diarrheic symptoms in children,^145^ possibly by favoring colonization and resolving dysbiosis. This could be explained by distinct adhesion proteins, conferring anti-diarrheic properties to *B. clausii* UBBC-07 and its similar ENTPro.

Comparative genomic analysis of *L. fermentum* 3872 identified genes encoding putative mucus-binding proteins, collagen-binding proteins, and EPSs which all contribute to enhance probiotic function.^146^ *L. fermentum* 3872 was isolated from breast milk of healthy human female and contains multiple vitamin synthesizing genes and adhesion genes. This would allow this probiotic candidate to persist in the GIT competing for similar sites with pathogens and favor nutritional processes which would make it an ideal candidate for addition in infant formula.^146^

Finally, although we consider in this review one by one the adhesion factors that characterise a certain probiotic, a good number of probiotics or probiotic candidates have multiple distinct putative adhesion factors. Currently, in literature, most studies are genomic studies focused on identification rather than downstream function of these adhesins. Hence, to what extent each of adhesins contributes within a multi adherence factor system on a certain probiotic is yet to be attributed.

## 3. Evidence Of Involvement Of Adhesins In Probiotic Function By Isolation Or Knockout Of Adherence Factors

Probiotic mechanisms on the host GIT cells include cytoprotection, cell proliferation, cell nutrition, and synthesis of proteins with gene expression changes.^147^ These can contribute to biological functions such as intestinal epithelial cell homeostasis and innate immune signaling regulation. ^148,149^ A number of studies provide evidence that these effects are possibly attributable in part to probiotic adhesion capacity, using either knockout molecules or isolated specific adhesins. In Table 1 and below we provide instances of studies in which changes in probiotic adhesion molecules have led to loss of probiotic function. Alternatively, specific adhesion molecules have been isolated to show a specific probiotic effect. For example, in several studies immune-related signaling by cytokines on host cells was affected when from the interacting probiotic was removed a specific structural molecule responsible for adhesion. From a broad literature screen, this was noted especially for *Lactobacillus* strains (Table 1). Overall, there was no apparent association between a specific adhesion factor and a known immunological response. Below are shown a few studies undertaking this approach.

**Table 1.** **Modifications of adhesion structures affects the probiotic function**. Specific modification of surface structures on probiotics, hypothesised to be potential adhesins, affected GIT rewiring with respect to direct interaction, immune stimulation through inflammatory cytokines production and pathogens inhibition.

Probiotic bacteria	Adhesion factor	Method	Function affected and mechanisms	Ref
*Ligilactobacillus salivarius* REN	SLP CbpA	Deletion mutant	Reduced ability to colonise the human gut as an effect of a deletion mutation in SLP CbpA.	^119^
*Lacticaseibacillus rhamnosus* GG	SpaCBA pili	Purification of the SpaCBA pili	Direct interaction of SpaCBA pili glycans with immune cells (DCs) via the C-type lectin receptor DC-SIGN, a key functional PRR modulating cytokine responses.	^37^
*Lacticaseibacillus rhamnosus* GG	SpaCBA pili	SpaCBA pilus knockout mutant	Mutant increased IL-8 expression, possibly through lipoteichoic acid on bacteria that binds to TLR2 on IECs	^108^
*Lacticaseibacillus rhamnosus* GG	SpaC pili	Isogenic mutant that lacks SpaC pili protein	Decreased IECs adhesion, cell proliferation and protection against intestinal injury by radiation by isogenic mutant.	^89^
*Lacticaseibacillus rhamnosus GG*	SpaCBA pili	Pilus-deficient LGG strain	Increased immune cell (NK cell) activity and no change in fecal microbial genus *Parabacteroides.*	^109^
*Bacteroides fragilis* NCTC 9343	Polysaccharide A (PSA)	Mutant lacking PSA, Purified PSA	Mutant: Failure to prevent disease and pro-inflammatory cytokine production in colon. PSA *In vivo*: Is needed to suppress pro-inflammatory IL-17 production by intestinal immune cells. PSA *In vitro*: Inhibits IL-1β induced inflammation on IECs.	^47–49^
*Bacteroides fragilis* (non-toxigenic)	Component of type VI secretion system (T6SS)	Deletion mutant	Mutant confers colonization resistance to enterotoxigenic *Bacteroides fragilis that is* associated with IBD, childhood diarrhea and colon cancer.	^150,151^
*Enterococcus Faecium* WEFA23	SLP	Removal of SLP by treatment with 5 mol/L LiCl, isolated SLP	Removal of SLP reduced adhesion capacity on pathogen *Listeria monocytogenes* CMCC54007. Isolated SLP decreases apoptosis of IECs induced by the pathogen.	^152^
*Lacticaseibacillus paracasei* (Lbp (LAP))	Surface-associated LAP from LbpLAP	LAP-expressing recombinant	Reduced ability of pathogen *Listeria monocytogenes* to adhere and invade as an effect of LAP-recombinant probiotic via receptor Hsp60 interaction to mammalian cells.	^153^
*Limosilactobacillus fermentum* MCC 2760 *(former*^16^ *Lactobacillus fermentum* MCC 2760)	Cell wall extract and crude bacteria culture filtrate	Extracts and conditioned media	Cell wall extraction induced the expression of IL-6, and crude culture filtrate enhanced the expression of IL-10 (anti-inflammatory cytokine).	^154^
*Lactobacillus rhamnosus* KL 53A and *Lactobacillus casei Fyos*	EPS	Enzymatic deglycosylation	Changes in the exopolysaccharide structure decreased adhesion efficiency of probiotic on IECs.	^155^
*Lactobacillus* *acidophilus* NCFM	SLP homolog PrtX	PrtX-deficient strain (gene deletion)	Prt-X deficient strain had increased adhesion to mucin and fibronectin. On antigen presenting cells (DCs) it increased induction of IL-6, IL-12 (pro-inflammatory) and IL-10 (anti-inflammatory), but IL-10/IL-12 ratio, (measure of the balance between pro-inflammatory and anti-inflammatory states) was higher.	^42^
*Lactobacillus* *acidophilus* NCFM	SLP A	Knockout mutant lacking SLP, purified SLP protein	Wild type bacteria adhered to DCs and consequently anti-inflammatory IL-10 production was higher with higher doses. Knockout bacteria for SLP A protein reduced binding to DCs (antigen presenting cells).	^41^
*L. Helveticus* MIMLh5 and NS8	SLP	Removal of SLP by treatment with 5 mol/L LiCl, isolated SLP	Both probiotic and isolated SLP showed similar mechanism of action via TLR2. Dampening of production of IL-10 was observed with probiotic stimulation but not isolated SLP.	^23^,^39^
*B. bifidum* ATCC 15696	Sialidase	Mutant of Siabb2 sialidase	Mutant decreased adhesion to IECs and mucin relative to the wild-type strain. SiaBb2 engages HMOs and mucin sialic acid for metabolic purposes and may facilitate *Bifidobacterial* adhesion.	^156^

Abbreviations: IL, Interleukin; SLP, Surface layer protein; EPS, Exopolysaccharide; PRR, Pattern recognition receptor; TLR, Toll like receptor; DCs, Dendritic cells; NK cell, Natural killer cell; IECs, Intestinal epithelial cells (e.g. Caco-2 cell line); IBD, Inflammatory Bowel Disease; LPS, Lipopolysaccharide; LiCl, Lithium Chloride; HMOs, Human milk oligosaccharides.

### 3.1 Isolated SLP from *Lactobacillus helveticus*

SLPs on *Lactobacillus helveticus* were defined decades ago as a layer of non-glycosylated protein in the bacterial cell wall.^157^ SLPs are thought to arbitrate a number of effects on host inflammatory mediators, innate immune signaling as well as in IECs homeostasis. A RCT with a parallel design, administering among others *L. helveticus* R0052 to healthy 3–12-month-old infants suggested an anti-inflammatory profile of this probiotic. The *L. helveticus* R0052 arm of this study showed an increase of the tumor necrosis factor alpha (TNF-α)/IL-10 ratio but no changes in fecal microbial composition.^158^ Interestingly, another *L. helveticus* (the NS8), pre-selected for its adhesion and survival properties, was able to diminish the pro-inflammatory effects of LPS by inducing higher levels of IL-10 in a macrophage cell line.^39^ This mechanism was possibly through SLP- mediated adhesion, however, when investigating the purified SLP on this strain it did not affect IL-10 (Figure 1). ^39^

Isolated SLP from *L. helveticus* MIMLh5 and the bacterium itself, were investigated using *in* *vitro* and *ex vivo* models. The two triggered an innate immune response via expression of pro-inflammatory TNFα and COX-2 in a human macrophage cell lines via recognition by the TLR2. No effect on anti-inflammatory IL-10 by isolated SLP was shown in this study (Figure 1).^23^ In addition, anti-inflammatory effects were proposed by reducing the activation of NF-ƙB in IEC lines.^23^

Both isolated SLP on *L. helveticus* SBT2171 as well as the probiotic bacteria itself induced antimicrobial peptide hBD2 expression in the host IECs via TLR2.^38^ This study shows that isolated SLPs present in other *Lactobacilli* have a similar stimulatory effect and proposes it as a common feature of a number of *Lactobacillus* species. Given that the TLR2 signaling is an important pathway for host protection against infections,^159^ isolated SLPs from *L. helveticus* could be considered for a supportive administration as probiotic molecules during human infant infections. Attempts have been made to use *L. helveticus* in infant infections. A multicenter clinical trial found that *L. helveticus* R0052, in combination with *L. rhamnosus*, did not prevent the development of gastroenteritis in 3–48-month-old children.^160^ Others found that the same probiotic showed higher fecal IgA levels in 3.5-6-month-old healthy infants ^40^ (Figure 1). However, *L. helveticus* R0052 in the second RCT was used in combination with two species of *Bifidobacterium* probiotics.

In summary, the role of both isolated SLPs on *L. helveticus* as well as *L. helveticus* bacteria alone have been directly compared and act through common pathways (i.e. TLR2). However, while the whole bacteria induced anti-inflammatory responses via dampening of e.g. IL-10 production this seems to be lost when using the purified SLPs^23,39^ (Figure 1), possibly due to a weaker effect of the adhesin or inadequate dose. Thus far, no RCT or *in* *vivo* human study has investigated the effect of isolated SLPs. A few RCTs using combination probiotics have administered *L. helveticus* probiotic bacteria, but do not answer whether the effect of the study was due solely to *L. helveticus* strain or its SLPs.

### 3.2 EPS-knockout mutant of *Lacticaseibacillus rhamnosus*

Besides its surface pili, the prototypical probiotic LGG contains exopolysaccharides (EPSs) shown to be involved in adhesion hence probiotic function. EPSs protect LGG against anti-microbial peptides in the GIT (Figure 1).^46^ This was confirmed when the EPS LGG mutant exhibited a decreased persistence in the murine GIT and was more sensitive to the host´s innate defense mechanisms.^46^ Interestingly, EPSs were also shown to change in the presence of different GIT conditions and despite different EPS molecules having similar mechanism of actions, their chemical structure seem to be sensitive to very small changes.^161^ LGG bacteria EPS-mutant and isolated EPS molecules were shown to interfere with Candida infection compared to LGG wild-type bacteria,^45^ suggesting that EPSs could confers anti-fungal properties. However, the study was performed in vaginal epithelial cells. Attempts have been made to study *Lacticaseibacillus rhamnosus* prevention of rectal colonization with Candida. A RCT of 150 12-year-old children on broad spectrum antibiotics were administered a mixture of probiotics containing among others *Lacticaseibacillus rhamnosus* and saw a decrease of prevalence of the fungal infection.^162^

In conclusion, LGG is a complex multifaceted probiotic with pili adhesion factors that appear to be important for immune mechanisms. EPS effects were shown by creating bacterial mutants lacking EPSs. However, although EPSs are relevant for bacteria persistence in the human GIT, evidence is unclear whether isolation of EPSs as single molecules is possible and which disease can benefit from it. Candidiasis infection caused by broad spectrum antibiotics is a clinical problem in children. And since administration of antibiotics is more often accompanied with intervals of probiotics in children, finding an isolated adhesin with mechanisms to both protect from antibiotic-induced candidiasis as well as diarrhea would be ideal.

### 3.3 Sialidase-knockdown mutant of *Bifidobacterium bifidum*

Interaction of some *B. bifidum* strains with GIT mucosa is mediated via the bacterium extracellular sialidase domain. Sialidases are proteinic adhesins involved in probiotic-mucosal interactions. Through enzymatic activity sialidases process a variety of carbohydrates such as human milk oligosaccharides (HMOs) that are needed for the bacteria self-metabolism and to promote *Bifidobacterial* growth. HMOs are a glycan source for the infant GIT microbiota.^156,163^ Hence HMO are clinically relevant to infant homeostasis in addition to *B. bifidum* itself being part of the dominant colonizers of the breast-fed infants GIT.^164^

Studies using *B. bifidum* ATCC 15696 mutant in sialidase domain (Siabb2) showed a decreased adhesion to human IECs and porcine mucin relative to the wild-type strain.^156^ This suggests a key role of sialidases as adhesins. Another *B. bifidum, B.bifidum* PRL2010, seems to targets host-derived glycans in mucus for nutrient attainment.^165^ This catabolic process, conserved in various strains, is an important colonization factor for *B. bifidum*.^165^

In summary, sialidase adherence activity began to be shown by usage of a bacteria mutant lacking this protein and could be the mechanism through which the bacteria anchors on the GIT mucosa. More studies are needed to elucidate the role of sialidases in isolation with respect to colonization of commensals, immunity and infections. Since sialidases are need for self-colonization of bacteria, studies with a mutant overexpressing the protein ought to be performed to investigate dose response. Hence, re-colonization with this probiotic via sialidases could contribute to resolving dysbiosis and protecting infants who experience decreased abundance of *B. bifidum* during GIT diseases.

## 4. Adhesion Of Probiotics And Pathogen Inhibition

Probiotic action on enteric pathogens has been widely studied. Mechanisms such as inhibition of colonization by competitive exclusion or secretion of antimicrobial substances are direct (physical) mechanism on pathogens, while acting on the epithelium and immune component affects indirectly pathogens.^166^ During competitive exclusion probiotics compete with pathogens for the same host mucosal receptors or for the same nutrition sources.^167^ This probiotic mechanism is potentially due to specific adhesion factors, like those of pathogens, as sites of adhesions on IECs and immune cell receptors binding.

*Lactiplantibacillus plantarum* DM 69 through its purified antimicrobial factors may competitively exclude intestinal *Salmonella enterica, as* shown by adhesion studies in an HCT-116 cell line.^168^ In addition, *L. plantarum* L15 strain was successfully established for prevention of pathogenic *Escherichia coli* adhesion.^167^ However, although the two *L. plantarum* strains showed potential for pathogen-inhibition and anti-microbial factors production, the responsible adhesion factors are still not well characterized. The studies speculate that this is potentially due to the adhesion analogs between probiotic and pathogens.

In another study, a novel probiotic mechanism involving mucus-binding peptides of LGG was shown to outcompete *Enterococcus faecium* colonization.^90^ Vancomycin-resistant enterococci peptides with known pathogenic properties were shown to share sequences with the peptides of SPCA-SRIP1 pili of the probiotic LGG.^90^ Hence, immunological and functional similarities between LGG and the pathogen *E. faecium* strain E1165 opens new frontiers for prophylaxis and treatment of vancomycin-resistant enterococcus infections.^90^ Supplementation of another *L. rhamnosus, Lacticaseibacillus rhamnosus* 19,070–2, to infants with intestinal colic was found to decrease crying and fuss time.^169^ These benefits may be due to the fact that probiotic bacterial pili can better adhere, colonize (Box 1) and exclude gas-forming *Clostridioides* (previously *Clostridium*) *difficile, Klebsiella pneumoniae*, and *Escherichia* which are increased in colic.^169,170^

Further mechanisms have been proposed for probiotics during enteric infections. Supplementation of probiotic *Streptococcus faecium* (i.e. *Enterococcus faecium*) and *Bacillus subtilis* during *Helicobacter pylori* infections diminished antibiotic-induced dysbiosis.^171^ This effect contributes to the *H. pylori* eradication success rate because it can restrict the growth of GIT antibiotic-resistant bacteria. On the other hand, *Enterococcus faecium* WEFA23 was shown to use the adhesion factor SLPs to inhibit five pathogens, and particularly *Listeria monocytogenes* CMCC54007.^152^ Removal of the adhesion factor SLP on *E. faecium* WEFA23 significantly decreased its adhesion capacity, suggesting that the probiotic SLP adhesion factor is responsible for pathogenic exclusion.

During rotavirus infection, *Lactobacillus acidophilus* AD031 and *Bifidobacterium longum* BORI had an effect on the duration of the diarrhea while other effects were non-significant.^172^ The supportive effect of *L. acidophilus* species during infections in infants were demonstrated by giving low doses of *L. acidophilus* (subsp. *L. Gasseri*) while *E. faecium* and *B. infantum* were found to decreased the frequency of late-onset sepsis in pre-term newborn.^173^ Live or killed *L. acidophilus* bacteria retained similar benefits while reducing the incidence of necrotizing enterocolitis (NEC).^174^ These data suggest that anti-microbial properties of *L. acidophilus* species are due to bacterial structures, possibly surface adhesion markers that persist after the probiotic is killed rather than to live probiotic metabolites.

In conclusion, from evaluation of pathogen inhibition studies several generalizable points come to light. First, the ability of probiotic bacteria to affect or eradicate pathogens has been studied mainly for pathogenic bacteria rather than e.g. viral infections. Second, most of these studies have widely speculated that the pathogen inhibition effect is due to similarities between adhesion factors of the probiotic bacteria and the pathogenic bacteria. However, very few^152,153^ or no studies have shown which of these adhesins are responsible for the effect. Finally, the ability of a probiotic strain to eradicate a pathogen is clinically highly relevant in cases where the probiotic acts on an enteric bacteria that is antibiotic resistant,^90^ hence alternative approaches are needed to eradicate the pathogen. In addition, it has been shown that in infants the choice of antibiotics affects both the healthy microbiota and the anti-bacterial resistance.^175^ Hence, if we could find a way to shift the microbiota by using probiotics, e.g. by increasing the proportion of certain healthy strains, we could achieve a benefit on the outcome of antibiotic resitant infections.

## 5. Age Related To Probiotic Function And Adhesion

Unlike adults which have a mature fully developed GIT mucosa, infants have an immature GIT mucosa with an immune component that needs yet to establish tolerance to the external environment. Adults have a restricted macromolecular epithelial passage, while infants have a high endocytic capacity with enhanced passage of macromolecules and pathogens.^176^ Earlier microbiota evaluations of *Lactobacillus* in feces showed that the infant intestine is initially colonized by only a few different strains whereas in adults there is a complex pattern with a higher diversity of strains.^177^ Species such as *L. rhamnosus* and *L. casei/paracasei* were characteristic of adult feces, whereas *L. gasseri* and *L. salivarius* were common in infant feces.^177^ Metagenomics and microbiome studies have shown also that children have a different functional microbiome with higher relative abundances of *Bacteroides* in children compared do adults.^178^ On the other hand *Bifidobacteria* in human babies < 2 year old was shown to be higher than adults.^179^ Human microbiota abundances are age-specific, but with increase of age a more diverse microbial colonization takes place shaping into adulthood GIT where intestinal microbiota is presumably relatively stable both in health and disease.^180,181^

**Figure 2.**
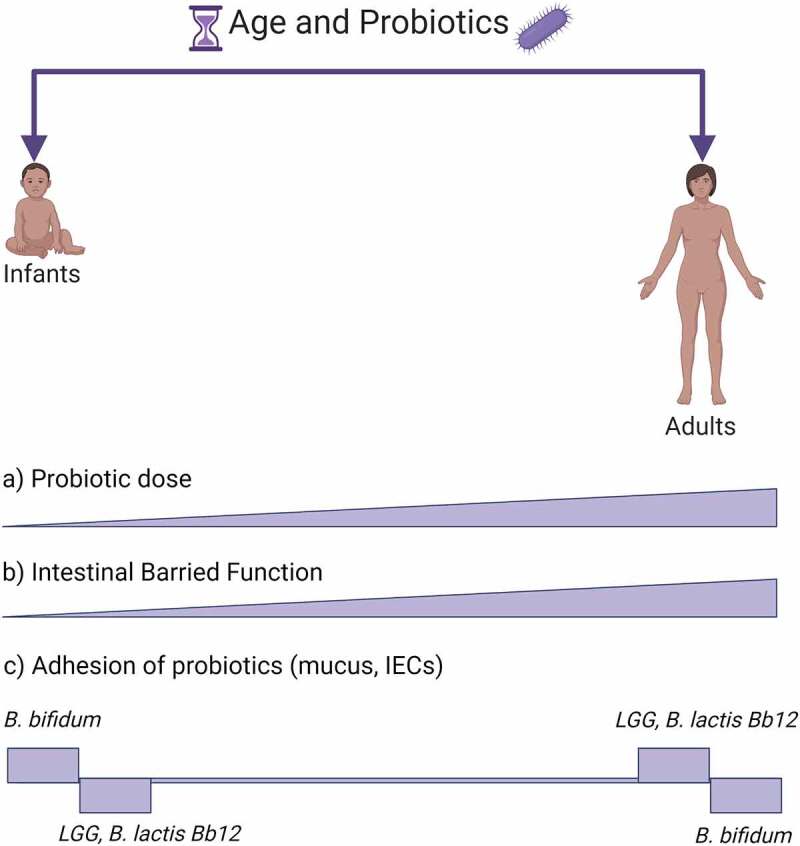
**Age and probiotics adhesion**. The figure shows a schematic view of probiotic and GIT characteristics changing with age. (a) Conventionally probiotics are given in increasing doses with age.^184^ (b) IECs, which are among the main cells interacting with probiotics in the GIT and form the intestinal barrier function, are less established early in life but with age they are progressively shaped into a fully functioning GIT barrier.^10,188^ Probiotic adhesion is strain- and age-dependent. In (c) are shown a few examples of probiotic strains, *B. bifidum*,^189^ *LGG*,^10,189^ *B. lactis Bb12*^189^, in which age-dependency has been investigated within a single study. Created with BioRender.com

Because of the age-specific nature of the GIT mucosa and microbiome, diseases affecting infants, children and adults require the use of tailored probiotics.^180^ Why a certain probiotic is used at a certain age hasn’t been systematically studied in literature. Nevertheless, administration of probiotic mixtures was shown to accelerate the transition into a mature, term-like microbiome which was *Bifidobacterium*-driven in preterm infants.^182^ Common GIT diseases affecting infants where probiotics are employed comprise necrotizing enterocolitis (NEC), infections, infant colic, use in preterm infants with the overall aim to improve short and long-term health.^183^ Children and adults employ probiotics mainly for acute gastroenteritis,^180^ acute diarrhea^184^ (e.g., antibiotic induced) and chronic disease such as inflammatory bowel disease (IBD), irritable bowel syndrome^185,186^ celiac disease and infections eradication.^187^ In Figure 2 and below are discussed a few probiotics strains in relationship to age.

Eminent strains, such as LGG, are used in pediatric and adult populations regardless of age. Antibiotic-associated diarrhea (AAD) is an acute side effect that occurs during most antibiotic treatments and where probiotics are regularly used. Although in most cases probiotics are used during antibiotic treatment, more studies are supporting a preventive use before antibiotic treatment.^190^ An elegant meta-analysis compared the current evidence of LGG use in AAD for children vs adults.^184^ The study indicated that LGG was effective in preventing diarrhea in both. However, while for children there was a link between dose and effect size, for adults such link did not show up.^184^ Why the later link did not show up is not clear, but possibly in the adult GIT LGG mechanism or adhesion and action might be different due to mucosal and microbiota differences with age. A methodological study showed that, immature IEC cell models HIEC-6 compared to the mature colonic Caco-2 were shown to react differently to LGG stimulation.^10^ HIEC-6 cells form a scarce barrier with lack of some tight junction compared to Caco-2 cells. LGG treatment could not improve the experimental barrier function in immature HIEC-6 cells.^10^ LGG employs a number of adhesion proteins (i.e. pili, EPS). Even thought LGG was able to prevent AAD in both pediatrics and adults, in neonates GIT mucus LGG adhesion was lower than adults^189^ hence the probiotic effect might be more a consequence of interaction with microbiota rather than strengthening the barrier function.^10^ Possibly, LGG might employ a dose-dependent effect in children that is more related to its adherence factor EPS that interacts with the microbiota and pathogens,^44,191^ rather than a direct interaction on IECs through pili adhesion factor.

A few studies have investigated age-dependent immune effects of probiotics mixtures in animals. Jeong et al compared the effects of a probiotic mixture (*L. casei, L. acidophilus, L. reuteri, B. bifidum*, and *S. thermophiles*) in young and aged rats to study age-dependent colitis.^192^ The mixture of probiotics seems to protect from LPS-induced inflammation, via NOS, COX2, TNF-α, IL-1β, CRP, and induce expression of intestinal barrier function markers (tight junctions, ZO-1, occluding) in an age-dependent manner. Kaushal et al showed that administration of probiotic Dahi consisting of Dahi bacteria along with *L. acidophilus* LaVK2 or *L. acidophilus* and *B. bifidum* BbVK3 improved age-related immune functions that are diminished with age. Peritoneal macrophage functions were enhanced by stimulating NOS and IL-6 and diminishing PGE2, and lymphocyte proliferation and IL-2 production was increased.^193^ It is interesting to note that, although both studies used a combination of probiotics, making it hard to attribute the effect to a single strain, both studies included *L. acidophilus* and *B. bifidum* in this combination. While the use of a mixture makes it hard to distinguish to which strain the effect is due, however, these studies are highly relevant for humans as pro-inflammatory markers such as TNF-a IL-6 and CRP have been shown to be associated both with ageing and are prototypical pro-inflammatory markers in the pathogenesis of IBD.^194,195^

Vast limitations exist when searching for studies on probiotics in relation to age. First, the few available intestinal cell line models are either fetal/neonatal-derived or adult-derived, from either humans and animals.^10,196^ There are no “in between” cell lines to represent children, and isolating primary cell lines from different pediatric ages merely for studying probiotic mechanisms seems unfeasible. Second, there seem to be universal strains used both in adults and pediatrics (e.g. *LGG, B. breve)* and strains unique to pediatrics *(B. infantis, L. rheuteri*), however why that is the case does not seem to be evidence-driven but rather historical, general health- and safety-driven.^197^ Third, a few studies directly comparing adhesion in infant and adult cell lines are general in referring to barrier function properties or mucus composition, and do not specifically discuss adhesins. Mechanistic studies comparing adhesion factors to age are not available and studies usually consider either infant or adult GIT.

## 6. Adhesion Factors As A Proxy To Predict The Effect On Probiotic Mechanism

**Figure 3.**
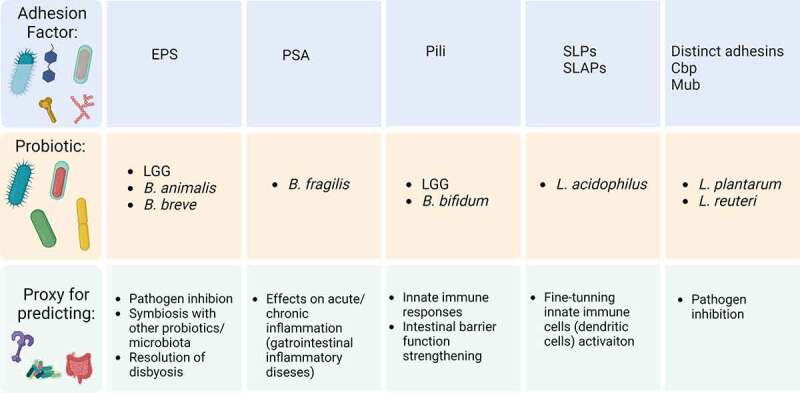
**Key adhesion factors as potential proxy for a** **global probiotic function.** Representation chart of some adhesion factors and potential application for global mechanisms with respective reference studies. First column: EPSs on LGG,^44–46^ *B. animalis*^63^ and *B. breve*.^67^ Second column: PSA on *B. fragilis*.^47–50^ Third column: Pili on *B. bifidum*^36,93^ and LGG.^37,106,108,109^ Fourth column: SLPs and SLAPs on *L. acidophilus*.^41–43,115^ Fifth column: distinct adhesins on *L. plantarum*^123,141^ and *L. reuteri*.^131^ Abbreviations: EPS, exopolysaccharides; LGG, *L. rhamnosus* GG; PSA, polysaccharide A; SLPs, surface layer proteins; SLAPs, surface layer associated proteins; Cbp, collagen binding protein; Mub, mucus-binding protein. Created with BioRender.com

It is suggested throughout the review that adhesion factors could be a proxy for probiotic mechanism and function. This idea is yet in its infancy given that adhesion factors are neither systematically studied nor formally classified in literature. However, a few important studies, discussed throughout the review and summarized in Figure 3 offer the strongest available evidence and may be considered as a starting point to suggest a specific adhesion factor as a proxy for a specific mechanism.

## 7. Concluding Remarks

This review aimed to gather knowledge on the pivotal role of adhesion factors in establishing a probiotic function in the GIT of adults and pediatrics. Numerous probiotic surface factors, defined here as adhesins, have shown promising results to be a proxy for predicting probiotic mechanisms in humans. However, only some of them (pili, SLPs, EPSs) are being widely investigated. Complex factors, related to both host and adhesion factors on bacteria, contribute to probiotic interactions with the host and these need to be understood individually before a systematic synthesis of this information. Possibly the age of the study population, probiotic dose, choice of the right *in* *vitro* models, the specific intestinal disease, and the individual variability of resident microbiota need to be considered in parallel to be able to interpret findings even for the same probiotic strain. Overall, observations made *in* *vitro*, in humans and animal models, on the role of probiotic adhesins, have great potential to guide probiotic function but are incomplete and can only be cautiously applied clinically in humans.

**Figure 4.**
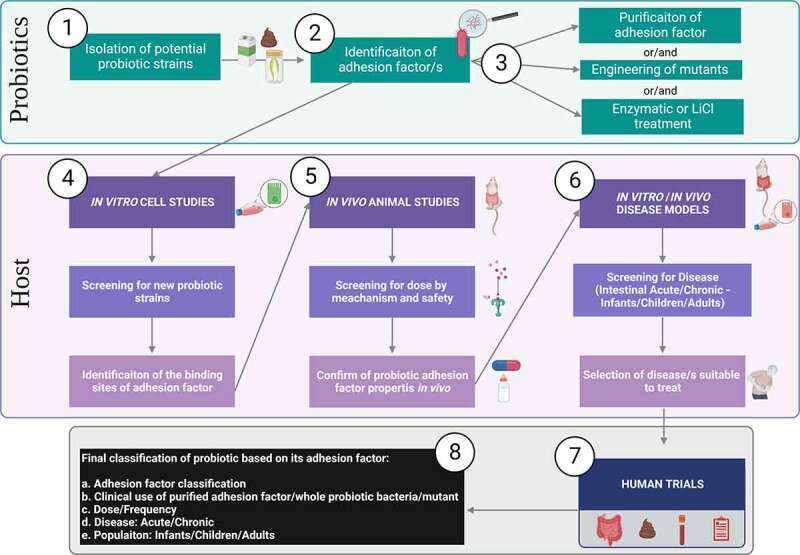
**Ideal step-by-step workflow for studies of adhesion factors role in probiotic function.** Given the mixed type of approaches found in literature the figure summarizes an ideal workflow for screening, experimental testing, and classification of probiotics considering adhesion factors. Bacteria from dairy, fermentation of fecal matter, etc, are isolated as potential probiotics (**Step 1**). Based on the bacteria type a hypothetical adhesion factor should be identified (**Step 2**). Then follows the purification of the adhesion factor (e.g. proteinic, polysaccharidic component), generation of mutants lacking, overexpressing the adhesion factor or enzymatic treatment of the bacteria (**Step 3**). *In vitro* studies, using most often IECs (e.g. Caco-2 cells) are the first experimental model used to investigate bacteria and adhesion factors for probiotic properties. Cell studies at this stage should ideally investigate the host receptors involved in the adhesion mechanism (**Step 4**). Once a probiotic and its adhesion mechanism have been identified, animal studies must aim to propose a dose range and mechanism of action *in* *vivo* (**Step 5**). Although adhesion factors are most often inert proteinic or complex carbohydrate by nature, hence considered safe, they could potentially be considered as drug-like when studying the metabolism *in* *vivo* to understand their half-life in the body. Once a preliminary hypothesis of the adhesin and healthy host is formulated, the next step should involve studies to decide which disease could benefit (**Step 6**). For instance, if it was hypothesized that an adhesion factor could benefit inflammatory conditions, established inflammatory models should be used to test this. Such models are for instance inflammation induced in IECs Caco-2 cell with inflammatory cytokines (e.g. IL-1β, TNF-α)^47^ or/and animal models such as dextran sulfate sodium (DSS)-induced colitis in animals.^11^ Next step comprises human trial conduction (**Step 7**). Although conduction of human trials in a stepwise manner takes longer, when possible, they should be performed in the following order: starting from pilot testing in healthy to study mainly safety, pilot trial in disease to mainly select a dose, and finally RCTs, Cross-Over or Parallel design trials with specific health outcomes. The 4 symbols represent samples used during human trials, intestinal biopsies, fecal samples, blood samples and symptoms questionnaires that can be used depending on the endpoint investigated and feasibility. Finally, considering both the probiotic and the host an adhesion factor-driven classification can be assigned to the probiotic (**Step 8**).

The few human clinical trials, using strains with well-characterized adherence factors, most often fail to consider the importance of adherence when a priori designing and discussing the study outcomes. We believe that a study design involving a bacterial mutant (e.g. genetic knockdown) and/or a purified/isolated adhesion factor is the best way to elucidate the role of an adhesin in probiotic function. For this purpose, more RCTs using clinically applied and probiotic candidates, are needed to assess the role of adhesins by using mutants or isolated adhesins in parallel with wild type strains. In Figure 4 we propose a step-by-step ideal planning of studies to systematically be conducted in order to reach a conclusion about a probiotic adhesin’s role and its clinical benefit.

Finally, although more systematic studies are needed, there is preliminary evidence that probiotic adherence factors contribute to and guide the overall probiotic function in health and disease. The adhesion mechanisms, and consequent effects on the host, need to be considered in a strain-specific manner when selecting a probiotic for clinical trials with the overall aim of applying tailored probiotic therapies in GIT diseases.

## Data Availability

Data sharing is not applicable to this article as no new data were created or analyzed in this study.

## Abbreviations

GIT Gastrointestinal tract

IECs Intestinal epithelial cells

SLP Surface layer protein

EPS Exopolysaccharide

LGG *Lacticaseibacillus rhamnosus* GG

IL Interleukin

NEC Necrotizing enterocolitis

RCT Randomized controlled trial

TLR Toll-like receptor
